# Dietary β-1,3/1,6-Glucan from Baker’s Yeast Supports Upper Respiratory Mucosal Immune Health in Healthy Adults: Evidence from a Randomized, Double-Blind, Placebo-Controlled Trial

**DOI:** 10.3390/nu18060961

**Published:** 2026-03-18

**Authors:** Takashi Kanno, Ken-Ichi Ishibashi, Shota Kajiyama, Takanori Ikawa, Taiki Morita, Kenta Murata, Shuu Imai, Machiko Nishioka, Yoshiyuki Adachi

**Affiliations:** 1 Laboratory for Immunopharmacology of Microbial Products, Tokyo University of Pharmacy and Life Sciences, Hachioji 192-0392, Japan; kannotak@toyaku.ac.jp; 2 Laboratory of Host Defense and Responses, Faculty of Nutrition, Kagawa Nutrition University, Sakado 350-0288, Japan; ishibashi.kenichi@eiyo.ac.jp; 3 R&D Headquarters, Life Science Research Department, Kobayashi Pharmaceutical Co., Ltd., Ibaraki 567-0057, Japan; s.kajiyama@kobayashi.co.jp (S.K.); tak.ikawa@kobayashi.co.jp (T.I.); tai.morita@kobayashi.co.jp (T.M.); k.murata@kobayashi.co.jp (K.M.); sh.imai@kobayashi.co.jp (S.I.); m.nishioka@kobayashi.co.jp (M.N.)

**Keywords:** dietary polysaccharides, functional polysaccharide, β-1,3/1,6-glucan, human health, mucosal immunity, randomized controlled trial

## Abstract

**Background/Objective**: Dietary polysaccharides are increasingly recognized as functional nutritional components that support human health through modulation of immune function. However, clinical evidence linking their intake to site-specific upper respiratory mucosal immune health in humans remains limited. This study investigated whether dietary β-1,3/1,6-glucan (SC-BG) from baker’s yeast may support upper respiratory mucosal immune health in healthy adults. **Methods**: Following in vitro assays on human dendritic cells (DCs), a randomized, double-blind, placebo-controlled parallel-group trial was performed in healthy adults (18–69 years) who consumed either SC-BG or placebo capsules for 12 weeks in Japan. The primary outcome was circulating DC activation. Secondary outcomes were mucosal immune markers including secretory immunoglobulin A (s-IgA) and lysozymes from site-specific mucosal swabs (posterior oropharyngeal wall/nasopharynx) and salivar, and self-perceived upper respiratory tract symptoms. **Results**: SC-BG increased CD80 expression in DCs in vitro. In the clinical trial, 40 participants were randomized (n = 20 per group), and 39 (SC-BG: n = 19, placebo: n = 20) were analyzed. At week 8, the SC-BG group showed higher cDC1 CD80 expression than placebo. SC-BG intake also attenuated declines in s-IgA levels in the posterior oropharyngeal wall and nasopharynx and increased salivary lysozyme concentrations. Participants receiving SC-BG reported fewer cumulative days with upper respiratory tract-related local and systemic symptoms. No test food-related adverse events were observed. **Conclusions**: These findings provide preliminary clinical and mechanistic observations suggesting that dietary SC-BG may enhance circulating cDC1 activation and may help support upper respiratory local mucosal immune health in healthy adults, highlighting the potential of dietary polysaccharides as functional nutritional strategies for maintaining immune resilience.

## 1. Introduction

Upper respiratory tract infections (URTIs) are one of the most frequently treated conditions in outpatient clinics worldwide, with an estimated annual economic burden exceeding 22 billion USD [1]. Adults experience approximately 2–3 episodes per year, whereas children may experience up to 8, resulting in substantial economic impact [2,3,4,5]. Typical symptoms encompass nasal symptoms (rhinorrhea and nasal congestion), pharyngeal symptoms (sore throat), and signs of lower respiratory involvement (cough and sputum) [6]. In particular, throat discomfort has been reported to reduce not only physical functioning but also health-related quality of life in Japan [7]. Even healthy individuals can temporarily experience oral and pharyngeal discomfort in low-humidity environments [8,9,10]. Indoor air often becomes dry during winter, and office workers frequently report temporary discomfort, such as oral and pharyngeal dryness, which negatively impacts their quality of life [11].

In recent years, increasing everyday immune resilience has attracted attention amid global infectious disease outbreaks, such as COVID-19 [12]. Dendritic cells (DCs), which function in both innate and adaptive immunity, play a central role in activating the immune response and are essential for defending against infections such as URTIs [13]. Human DCs consist primarily of two subtypes: conventional dendritic cells (cDCs) and plasmacytoid dendritic cells (pDCs) [14]. Human cDCs are derived from bone marrow progenitors and circulate in the blood to the spleen and lymph nodes, where they constantly interact with T cells [15]. cDCs exhibit a variety of pattern recognition receptors (PRRs), including Toll-like receptors (TLRs) and C-type lectin receptors, whereas pDCs predominantly express TLR7 and TLR9. cDCs possess stronger antigen-presenting abilities than pDCs [16,17,18]. cDCs phagocytose pathogens, degrade them into peptides, and cross-present them on MHC-I. Upon cross-presentation, the expression of CD80/CD86 increases and these molecules bind CD28 on CD8^+^ T cells, promoting the generation of cytotoxic T lymphocytes (CTLs), which mediate antiviral immunity [15,16,17]. Moreover, cDCs induce Th17 cells by producing IL-23, contributing to the defense against fungi and extracellular bacteria [18]. CD4^+^ T cells activate B cells through MHC-II, promoting their maturation into IgA-producing plasma cells. Secretory IgA (s-IgA) plays a central role in mucosal host defense [19,20,21].

β-1,3-D-glucan (BG) is an essential polysaccharide found in the cell walls of most fungi, some bacteria, and both higher and lower plants. In mammals, fungal BG is recognized by the host immune system as a pathogen-associated molecular pattern and binds to the host’s C-type lectin receptor Dectin-1, inducing immune processes, including inflammation and phagocytosis [22,23,24,25]. The food used in this clinical study contained more than 80% β-1,3/1,6-glucan derived from baker’s yeast and has been reported to reduce upper airway inflammation, a symptom of influenza, by modulating immune function [26].

However, despite growing evidence for the immunomodulatory effects of yeast-derived β-glucans, it remains unclear whether oral intake of baker’s yeast-derived β-1,3/1,6-glucan can modulate human dendritic cell activation and upper respiratory mucosal immune markers in vivo, and whether such changes are accompanied by reductions in subjective throat discomfort and URTI-related symptoms in healthy adults. Accordingly, the primary objective of this randomized, double-blind, placebo-controlled trial was to evaluate the effects of oral intake of baker’s yeast-derived β-1,3/1,6-glucan on dendritic cell activation, upper respiratory mucosal immune markers, and self-reported throat discomfort/URTI-related symptoms in healthy adults.

## 2. Materials and Methods

### 2.1. In Vitro Experiments

#### 2.1.1. Study Materials

In this study, β-1,3/1,6-glucan derived from *Saccharomyces cerevisiae* (“baker’s yeast”) was used, referred to as SC-BG, specifically the commercial product Wellmune^®^ (Kerry Inc., Beloit, WI, USA).

#### 2.1.2. In Vitro Proliferation Assay

Peripheral blood mononuclear cells (hPBMCs) (Lonza, Basel, Switzerland) were used to isolate CD14-positive cells using the Pan Monocyte Isolation Kit (Miltenyi Biotec, Bergisch Gladbach, Germany). These cells were cultured for 5 days in LGM-3 lymphocyte growth medium (Lonza) supplemented with 50 ng/mL IL-4 (R&D Systems, Minneapolis, MN, USA) and 50 ng/mL GM-CSF (R&D Systems) to induce differentiation into conventional dendritic cells (cDCs). The differentiated cDCs were seeded at 1 × 10^5^ cells/well into 48-well flat-bottom plates, then stimulated with poly(I:C) (25 µg/mL; InvivoGen, San Diego, CA, USA) or SC-BG (10, 30, and 90 µg/mL) and cultured at 37 °C with 5% CO_2_ for 40 h. The concentrations of SC-BG were established using a three-fold serial dilution series based on preliminary pilot studies. After culture, the cells were stained with the following antibodies: FITC–anti-human CD11c (BioLegendm, San Diego, CA, USA), PE–anti-human HLA-DR (BioLegend), APC–anti-human CD40 (BioLegend), and APC–anti-human CD80 (BioLegend). Samples were analyzed using a BD Accuri C6 Plus cytometer (BD Biosciences, San Jose, CA, USA), and flow cytometry data were analyzed using FlowJo Software v10.2 (BD Biosciences). The gating strategy of the flowcytometry is shown in Supplementary Figure S1.

For the assessment of TNF-α production, hPBMCs were seeded at 1 × 10^6^ cells/well in 24-well flat-bottom plates and incubated with poly(I:C) or SC-BG for 48 h at 37 °C with 5% CO_2_. Supernatants were collected, and TNF-α concentrations were quantified using a Rebis Human TNF-α ELISA Kit (Fujifilm Wako Pure Chemical Corp., Osaka, Japan).

#### 2.1.3. Dectin-1 Binding Assay

A variety of β-glucan and polysaccharide preparations were assessed, including SC-BG, glucan from baker’s yeast, zymosan A from *S. cerevisiae*, β-D-glucan from barley, cellulose, β-1,3-glucan from *Euglena gracilis*, laminarin, curdlan (Sigma-Aldrich, St. Louis, MO, USA), depleted zymosan, zymosan (InvivoGen), and fucoidan (Fujifilm Wako, Osaka, Japan). Each sample was suspended in PBS at 5 mg/mL, dispersed or solubilized via ultrasonic treatment (50% amplitude, 30 s; UD-100, TOMY SEIKO, Tokyo, Japan). The preparations were stored as stock solutions until use.

For measurement of Dectin-1 binding to insoluble fractions, stock suspensions were diluted to 1 mg/mL, centrifuged at 1500× *g* for 5 min, and subsequently washed three times with PBS. Following blocking with 2% FBS–PBS for 20 min at 4 °C, samples were transferred to 96-well V-bottom plates and incubated with biotinylated mouse Dectin-1 Fc fusion protein (mDectin-1; 2 µg/mL, prepared as described previously [27]) and streptavidin-PE (1 µg/mL; BioLegend) for 30 min at 4 °C. Samples were washed and analyzed using a FACS Celesta cytometer (BD Biosciences). The data were processed using FlowJo v10.0.0. Flowcytometry particle isolation is shown in Supplementary Figure S2.

For measurement of Dectin-1 binding to soluble fractions, stock suspensions were diluted to 100 µg/mL, centrifuged at 5000× *g* for 10 min, and the resulting supernatant was tested. Wells of a 96-well half-area plate (Greiner Bio-One, Kremsmuenster, Austria) were coated with mDectin-1 (1 µg/mL in 0.1 M phosphate buffer, pH 6.8) overnight at 4 °C, subsequently blocked with 1% BSA–PBS, and incubated with samples for 1 h. The plates were sequentially incubated with biotinylated mDectin-1 (500 ng/mL) and streptavidin-HRP (BioLegend), followed by TMB substrate (Seracare, Milford, MA, USA). Absorbance was measured using a Multiskan FC plate reader (Thermo Fisher Scientific, Waltham, MA, USA). Soluble binding was quantified using laminarin from a different lot than that used in the samples as the reference standard.

#### 2.1.4. Statistical Analysis

Normality was assessed using the Kolmogorov–Smirnov test. If normality was confirmed, data were analyzed by one-way ANOVA followed by Dunnett’s post hoc test. If normality was not confirmed, Steel’s test was used instead. Statistical significance was set at *p* < 0.05.

### 2.2. Clinical Study

#### 2.2.1. Test Foods

Two types of capsules were prepared by Kobayashi Pharmaceutical Co., Ltd. (Osaka, Japan): one containing SC-BG and the other a placebo without SC-BG. The raw material, SC-BG (Wellmune F3005; Lot No. 2010193), was purchased from Kerry Group. The SC-BG capsules were formulated so participants would consume 180 mg/day of baker’s yeast-derived β-1,3/1,6-glucan. The placebo capsules were identical, except for the absence of SC-BG. The nutritional content per daily intake is presented in Table 1. Participants consumed two capsules per day (one dose daily) for 12 weeks. The appearance and flavor of both capsule types were indistinguishable, which was confirmed by the ethics committee.

Comprehensive quality control and safety assessments were performed on both the raw material and the final capsules. Allergen testing (covering egg, milk, wheat, buckwheat, peanut, and shellfish) was conducted by FALCO biosystems Ltd. (Kyoto, Japan). Additionally, mycotoxin analysis (total aflatoxin and aflatoxins B1, B2, G1, and G2), residual pesticide screening (815 compounds), and radioactivity analysis (Iodine-131, Cesium-134, and Cesium-137) were performed by Q’SAI Analytical Research Institute Co., Ltd. (Fukuoka, Japan). All tested parameters were below the detection limits.

#### 2.2.2. Participants

The study was conducted from December 2022 to May 2023 at the Medical Corporation Hokubukai Utsukushigaoka Hospital (Hokkaido, Japan). Participants were recruited via the “Hokuto Unity Club,” a membership-based clinical trial volunteer organization operated by the same institution, and through web-based advertisements. Eligible participants were healthy Japanese men and women aged 18–69 years who, based on self-reporting, perceived themselves as prone to colds and had experienced at least one episode of common cold-like symptoms within the previous three years. For the purposes of this study, a cold was defined as the presence of symptoms such as low-grade fever (<38 °C), runny nose, nasal congestion, sore throat, pain on swallowing, sneezing, cough, sputum, and general fatigue. Eligibility was assessed at screening using blood testing, and a physician interview. Only individuals who had not received COVID-19 or influenza vaccination since June 2022 and were not planning to receive either vaccine during the study period were included. The exclusion criteria were established either to ensure the appropriate conduct of the clinical trial or to protect participant safety. Specifically, criteria (1)–(5), (7), (10)–(14), and (16) were set to ensure the appropriate conduct of the trial, whereas criteria (6), (8), (9), (15), and (17) were set to protect participant safety. The exclusion criteria were as follows: (1) individuals who were currently receiving pharmacological treatment for any disease; (2) individuals who had, were being treated for, or had a history of severe disease, including diabetes, renal or hepatic disease, cardiac disease, thyroid disease, adrenal disease, or other metabolic disorders; (3) individuals with gastrointestinal disease or a history of gastrointestinal disease (except for appendectomy); (4) individuals who routinely consumed supplements intended to enhance immune function, including Agaricus, cordyceps, lion’s mane mushroom, reishi mushroom, cauliflower mushroom, fucoidan, propolis, or lactic acid bacteria marketed as beneficial for immune function; (5) individuals with chronic disease who regularly used medication; (6) individuals who were at risk of developing allergic symptoms to the test food (a β-glucan-containing food), or who were at risk of developing allergic symptoms to other foods or medications; (7) individuals who routinely used steroids or other agents considered likely to affect the test results; (8) individuals who had donated blood (or undergone equivalent blood collection) in excess of 200 mL within the previous month or 400 mL within the previous 3 months; (9) individuals with severe anemia; (10) individuals who tested positive on screening tests for infectious diseases, including syphilis, hepatitis B virus, hepatitis C virus, or human immunodeficiency virus (HIV); (11) individuals with a current or past history of drug dependence or alcohol dependence; (12) individuals with eating disorders; (13) individuals whose habitual alcohol consumption exceeded an average of 60 g/day of pure alcohol; (14) individuals who were likely to change their lifestyle during the study period (e.g., long-term travel or business trips lasting 1 week or longer); (15) individuals who were pregnant, breastfeeding, or possibly pregnant; (16) individuals who were currently participating in another human clinical trial, or who had participated in another human clinical trial within the previous 3 months; (17) individuals who were otherwise judged by the principal investigator to be unsuitable for participation in the study.

All the participants provided written informed consent prior to enrolment. The study protocol was approved on 1 December 2022, by the Utsukushigaoka Hospital Ethics Review Committee and registered with the UMIN Clinical Trials Registry (UMIN000049706). The study adhered to the 1975 Declaration of Helsinki (revised 2013) and the Japanese “Ethical Guidelines for Medical and Biological Research Involving Human Subjects” [28,29]. The sample size was based on previous findings of increased salivary s-IgA levels after SC-BG intake [30]. Assuming an effect size of 1.1, α = 0.05 (two-sided), 80% power, and accounting for dropouts 30% due to the 12-week intervention period during the winter season, the required sample size was set to 20 participants per group (total 40).

#### 2.2.3. Study Design

This was a randomized, double-masked, placebo-controlled parallel-group trial. The study consisted of screening, a 2-week pre-observation period, and a 12-week intake period. Participants completed a daily health questionnaire and underwent clinical evaluations at screening and at weeks 0, 8, and 12. The primary endpoint was evaluated at week 8. Secondary endpoints were assessed at week 8 and/or week 12, depending on the specific parameter. An independent allocator generated a computer-based stratified block randomization sequence using Microsoft Excel (block size: four), with age, body weight, baseline dendritic cell activity, and s-IgA levels as allocation factors. To ensure baseline comparability between groups, a forced randomization approach was implemented based on these factors. Allocation was concealed using sealed, opaque envelopes. All study personnel, investigators, and participants were masked to group allocation.

#### 2.2.4. Study Endpoints

Primary endpoint: Dendritic cell activation.

Secondary endpoints: Immune markers (s-IgA, Lysozyme, NK cell activity, neutrophil phagocytic activity, anti-β-glucan antibody titers), daily health questionnaire, quality of life (SF-8), and safety.

#### 2.2.5. Dendritic Cell Activation

Blood samples were collected at screening and week 8. Peripheral blood mononuclear cells were isolated using BD Vacutainer CPT tubes (BD Biosciences). The cells were cryopreserved in Cellbanker^®^ 1 (ZENOAQ, Koriyama, Fukushima, Japan) at –80 °C and subsequently stored in liquid nitrogen until analysis. Prior to cytometric analysis, PBMC samples were anonymized and assigned coded identifiers. Flow cytometry measurements were performed by laboratory personnel who were blinded to group allocation. Instrument settings were standardized before sample acquisition, and identical gating strategies were applied across all samples using predefined criteria to minimize operator-dependent variability. After thawing, the cells were washed, stained with antibodies (Supplementary Table S1), and analyzed using a FACS Celesta cytometer. cDC1 were defined as 7-AAD^−^, lineage ^−^, CD11c^+^, HLA-DR^+^, and CD141^+^. cDC2 were defined as 7-AAD^−^, Lineage^−^, CD11c^+^, HLA-DR^+^, and CD1c^+^(Supplementary Figure S3). Activation marker expression (CD40, CD80, and CD86) was quantified using the mean fluorescence intensity.

#### 2.2.6. Mucosal Immune Markers (Saliva and Swab Samples)

Saliva was collected at screening and at weeks 0, 8, and 12 using Oral Fluid Collector swabs (SOMA Bioscience Limited, Wallingford, UK). Additional saliva samples were collected using Salivette tubes for specific assays. Oropharyngeal and nasopharyngeal swabs were collected from the posterior oropharyngeal wall and the nasopharyngeal mucosa using FLOQSwabs^®^ (Copan diagnostic Inc., Murrieta, CA, USA) at weeks 0, 8, and 12.

Saliva s-IgA levels were quantified using an enzyme immunoassay test kit (Yanaihara Institute Inc., Fujinomiya, Shizuoka, Japan). Lysozyme in saliva was measured using the AssayMax Human Lysozyme ELISA Kit. s-IgA from oropharyngeal swabs was analyzed using a human s-IgA ELISA (Yanaihara Institute Inc). Nasopharyngeal s-IgA was quantified and corrected for total protein concentration (NanoOrange Protein Quantitation Kit, Thermo Fisher Scientific). Anti-β-glucan IgA and IgG titers were measured in saliva, oropharyngeal, and nasopharyngeal samples at the designated time points using previously described ELISA methods [31,32].

#### 2.2.7. Systemic Immune Markers (Blood Samples)

Blood samples were collected at weeks 0 and 12. These samples were analyzed for the following systemic immune markers by Sapporo Clinical Laboratory Inc. (Hokkaido, Japan): total IgA, Natural Killer (NK) cell activity, Neutrophil Phagocytic Activity, C-Reactive Protein (CRP), and anti-β-glucan IgA and IgG titers.

#### 2.2.8. Daily Health Questionnaire and QOL (SF-8)

Participants recorded daily symptoms for the following:

Runny nose, nasal congestion, sore throat, pain on swallowing, sneezing, cough, sputum, general fatigue, muscle pain, joint pain, headache, loss of appetite, and chills. Symptoms were scored on a 0–3 scale (none to severe). The SF-8 survey was administered at weeks 0, 8, and 12 [33].

#### 2.2.9. Safety Evaluations

Safety was assessed in the safety set, defined as all participants who ingested the test food at least once, through physician examinations, vital signs, and laboratory tests (hematology, biochemistry, and urinalysis) at screening and at weeks 0, 8, and 12. Adverse events were evaluated by the study physician, who determined their occurrence and clinical relevance by considering whether observed findings or changes were within the range of normal day-to-day variability, based on physical examination findings, vital signs, and laboratory data.

#### 2.2.10. Statistical Analysis

Analyses were performed using Microsoft Excel, EZR v1.54, and IBM SPSS v26. Statistical significance was set at *p* < 0.05. Normally distributed variables were compared using independent *t*-tests, and non-normally distributed variables were compared using Wilcoxon rank-sum tests. Within-group comparisons were performed using paired *t* tests. Cumulative symptom days were compared using chi-square tests. SF-8 scores were analyzed using the Wilcoxon test. Safety parameters were evaluated using chi-square and one-sample *t* tests.

## 3. Results

### 3.1. In Vitro Assay

#### 3.1.1. Activation of cDCs

**Figure 1:**
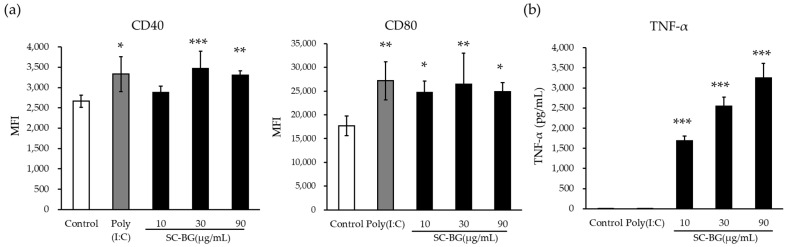
SC-BG-mediated activation of human dendritic cells. (**a**) Expression levels of CD40 and CD80 on human monocyte-derived cDCs following stimulation with SC-BG for 40 h, presented as mean fluorescence intensity (MFI). (**b**) TNF-α concentrations in hPBMC culture supernatants after 48 h of stimulation with SC-BG. Data are expressed as mean ± SD (*n* = 5). Experiments were performed in triplicate for (**a**) and in duplicate for (**b**) to ensure reproducibility. Statistical significance was determined using one-way ANOVA followed by Dunnett’s test for (**a**) and Steel’s test for (**b**). Statistical significance vs. control is indicated as * p < 0.05, ** p < 0.01, and *** p < 0.001. SC-BG, β-1,3/1,6-glucan from *Saccharomyces cerevisiae*; cDCs, conventional dendritic cells; MFI, mean fluorescence intensity.

To evaluate the ability of SC-BG to activate dendritic cells, CD14^+^ monocytes were isolated from hPBMCs and differentiated into cDCs (CD11c^+^, HLA-DR^+^). The expression of the activation markers CD40 and CD80 was measured after stimulation with SC-BG (Figure 1a). SC-BG significantly increased CD40 expression at concentrations ≥30 µg/mL and CD80 expression at concentrations ≥10 µg/mL. To further assess the immunostimulatory activity associated with cDC activation, TNF-α production by hPBMCs was quantified (Figure 1b). SC-BG induced a dose-dependent and statistically significant increase in TNF-α secretion compared to that in the vehicle control.

#### 3.1.2. Dectin-1 Binding Assay

To evaluate the ability of SC-BG to bind to Dectin-1, both insoluble particle binding and soluble fraction binding were assessed using a mouse Dectin-1 protein (Table 2). Polysaccharide reagents derived from *S. cerevisiae* were clearly separated into two groups based on their binding to insoluble particles via Dectin-1. SC-BG belonged to the high-binding group and showed Dectin-1 binding comparable to that of other yeast-derived β-glucan preparations with high activity. Compared with non-yeast-derived β-glucans, such as paramylon and curdlan, SC-BG exhibited markedly stronger Dectin-1 binding. The soluble Dectin-1-binding capacity of SC-BG was comparable to that of other *S. cerevisiae*-derived preparations. However, the levels were much lower than those of laminarin, which is known to inhibit Dectin-1 activation through soluble competition. These findings suggest that SC-BG possesses strong Dectin-1-mediated activation potential.

### 3.2. Clinical Study

#### 3.2.1. Subjects

**Figure 2:**
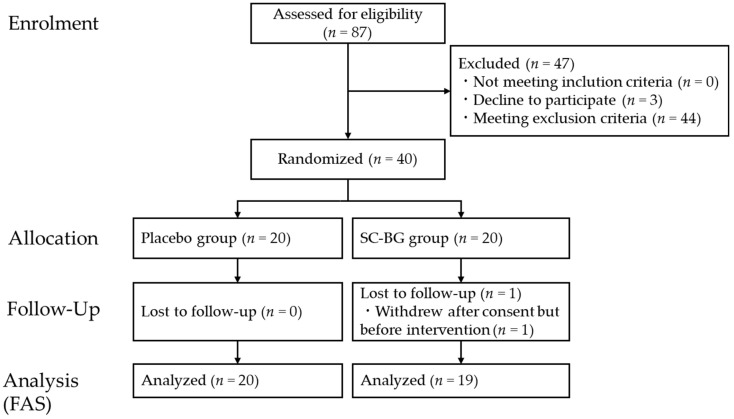
CONSORT diagram of this study.

Figure 2 shows the CONSORT diagram of participant enrolment and flow. A total of 87 volunteers were screened after providing informed consent (4–21 December 2022). Screening included standard blood tests, assessment of dendritic cell activity, and biochemical analyses. From these, 40 eligible participants were selected and randomized into two groups. One participant withdrew before consuming the test food; this individual was excluded from the analysis because no food intake or data collection occurred. Therefore, 39 participants completed the study (19 in the SC-BG group and 20 in the placebo group), and analyses were conducted using the Full Analysis Set (FAS). Intervention adherence was assessed using daily intake diaries and by counting returned capsules at the end of the intervention. Overall compliance was high, with a mean (SD) compliance rate of 99.2% (SC-BG: 98.7%; placebo: 99.7%). The baseline characteristics are shown in Table 3.

#### 3.2.2. Primary Endpoint: Dendritic Cell Activation

The mean expression levels of activation markers on DCs are shown in Table 4. After 8 weeks of intake, the SC-BG group exhibited a significantly higher CD80 expression level in cDC1 than the placebo group (*p* < 0.01). CD86 expression was not significantly affected by SC-BG. For CD40, the placebo group showed a significant decline after 8 weeks, whereas the SC-BG group demonstrated a slight, non-significant increase. In cDC2, neither CD40 nor CD80 levels differed significantly between the groups at week 8. CD86 levels increased significantly from baseline in the placebo group (*p* < 0.05), but no between-group differences were observed.

#### 3.2.3. s-IgA and Lysozyme

The results are summarized in Table 5. For salivary s-IgA, no significant changes were observed in the placebo group at weeks 8 or 12 compared with baseline. In contrast, the SC-BG group showed a significant increase in salivary s-IgA at Week 12. However, neither the raw values nor the change scores demonstrated significant between-group differences. For nasopharyngeal s-IgA, the placebo group exhibited a significant decrease at week 12 from baseline, whereas no significant decrease was observed in the SC-BG group. The change score at week 12 was significantly higher in the SC-BG group than in the placebo group (*p* < 0.05). For oropharyngeal (throat) s-IgA, the placebo group showed significant decreases at weeks 8 and 12. In the SC-BG group, no significant decrease was observed at week 8, although a decrease was detected by week 12. The week 8 change score was significantly higher in the SC-BG group than in the placebo group (*p* < 0.05). For salivary lysozyme, the raw concentration at Week 12 was significantly higher in the SC-BG group than in the placebo group (*p* < 0.01), although the change scores did not differ significantly between groups.

#### 3.2.4. IgA, NK Cell Activity, Neutrophil Phagocytosis, and CRP

No significant between-group differences were observed in the changes from baseline for serum IgA, NK cell activity, neutrophil phagocytosis, or CRP. Within-group analyses indicated that serum IgA levels significantly increased at week 12 in both groups (*p* < 0.05). Neutrophil phagocytic activity also significantly increased at week 12 in both groups (*p* < 0.05). No significant differences were detected between groups for any of these parameters. Full numerical results are presented in Table 6.

#### 3.2.5. β-Glucan Antibody Titers

The results of β-glucan-specific antibody titers are summarized in Supplementary Table S2. For anti-BG IgA in blood, no significant changes were observed in the placebo group, whereas the SC-BG group showed a significant decrease at week 8 (*p* < 0.05). However, no significant between-group differences were detected. For anti-BG IgG in blood, the placebo group exhibited a significant decrease at week 8 (*p* < 0.05), while no significant changes were observed in the SC-BG group; again, no between-group differences were noted. Salivary anti-BG IgA levels increased significantly at Week 8 in both groups (*p* < 0.01), with no differences between groups. In contrast, nasopharyngeal anti-BG IgA levels increased significantly in the SC-BG group at weeks 8 and 12 (*p* < 0.01), whereas no significant changes were observed in the placebo group; between-group differences were not significant. For oropharyngeal anti-BG IgA, the placebo group showed significant decreases at weeks 8 and 12 (*p* < 0.01), while no significant changes were observed in the SC-BG group. The change score at week 8 was significantly higher in the SC-BG group than in the placebo group (*p* < 0.05).

#### 3.2.6. Daily Health Questionnaire and QOL (SF-8)

Cumulative symptom days for each group are summarized in Table 7. Participants in the SC-BG group reported significantly fewer cumulative days with local symptoms at weeks 8 and 12, including runny nose, nasal congestion, sore throat, pain on swallowing, sneezing, cough, and sputum production. Systemic symptoms such as general fatigue, headache, loss of appetite (Week 8 only), and chills (Week 12 only) were also reduced in the SC-BG group. Joint pain was the only symptom for which the placebo group reported significantly fewer cumulative days. Regarding moderate-to-severe symptoms, SC-BG intake significantly reduced the cumulative days of nasal congestion, sore throat, pain on swallowing, sputum, and headache at weeks 8 and 12, as well as general fatigue at week 12. For severe symptoms alone, sore throat was significantly less frequent in the SC-BG group at both time points.

In the placebo group, Physical Functioning (PF) scores of SF-8 significantly declined at week 8 (*p* < 0.05). In contrast, the SC-BG group showed significant improvements in the Mental Component Summary (MCS) at week 8 (*p* < 0.01), along with improvements in Emotional Role Functioning (RE) and Mental Health (MH) at week 8, and sustained improvements in MH at week 12 (*p* < 0.05). Between-group comparisons demonstrated that, at week 8, the SC-BG group had significantly higher scores in several mental health-related domains compared with the placebo group (*p* < 0.01). Detailed SF-8 outcomes are provided in Supplementary Table S5.

#### 3.2.7. Safety Evaluation

There were no significant intergroup differences in the physical examination findings. Some laboratory parameters showed statistically significant changes; however, these remained within the normal clinical range. The study physician judged that none of these represented clinically meaningful issues. No adverse events attributable to the test foods were observed.

## 4. Discussion

In the present study, we investigated whether a dietary polysaccharide, β-1,3/1,6-glucan derived from baker’s yeast, may support upper respiratory mucosal immune health in healthy adults. By integrating mechanistic in vitro analyses with a randomized, double-blind, placebo-controlled human trial, we observed that dietary β-1,3/1,6-glucan intake was associated with the maintenance of secretory IgA levels in the posterior oropharyngeal wall and nasopharynx, increased salivary lysozyme concentrations, and a reduced burden of self-perceived upper respiratory tract symptoms. To our knowledge, these findings provide preliminary clinical observations suggesting that dietary polysaccharides may influence site-specific mucosal immune markers in the upper respiratory tract.

In vitro, SC-BG enhanced the expression of activation markers (CD40 and CD80) in human monocyte-derived dendritic cells and dose-dependently increased TNF-α production in hPBMCs. SC-BG exhibited a strong binding affinity for Dectin-1. Although receptor binding alone does not necessarily equate to downstream signaling, such particulate engagement is compatible with functional Dectin-1 activation via phagocytic synapse formation [25]. In the clinical trial, SC-BG intake significantly increased the activation of circulating cDC1 and maintained s-IgA levels in the throat and nasopharynx, as well as saliva lysozyme concentrations. Participants receiving SC-BG reported significantly fewer cumulative days of URTI-related local and systemic symptoms.

Yeast-derived β-1,3/1,6-glucan has been shown to activate dendritic cells via Dectin-1 and enhance both innate and adaptive immunity, particularly inducing downstream Th1 and Th17 responses that strengthen antiviral and antibacterial defense mechanisms [24,34]. In this study, SC-BG enhanced dendritic cell activity and increased TNF-α production. The upregulation of dendritic cell activation markers corresponds to maturation, improved antigen presentation, and enhanced co-stimulatory signaling to T cells [35]. Increased TNF-α production also contributes to dendritic cell differentiation and maturation following viral exposure [36]. These results suggest that SC-BG may modulate the immune system by activating dendritic cells via Dectin-1 engagement and promoting TNF-α-mediated pathways, which may contribute to the maintenance of upper respiratory mucosal immune health and to the alleviation of URTI-related symptoms in healthy adults.

In the clinical trial, SC-BG intake significantly increased CD80 expression in cDC1 and significantly reduced the incidence of URTI-related symptoms. Dendritic cell subsets include cDC1, cDC2, and pDC. Among these, cDC1 excels at recognizing foreign antigens, performing cross-presentation, activating CD8^+^ cytotoxic T lymphocytes (CTLs), and inducing Th1 responses, all of which are critical for antiviral defense [24,36,37,38,39,40,41]. A deficiency of cDC1 impairs CD8^+^ T-cell responses and reduces IFN-γ and TNF-α production, resulting in weakened resistance to viral and fungal infections [37,38]. Therefore, SC-BG intake may enhance protection against infections by promoting interactions between cDC1 and T cells and increasing CTL responses [40,41]. To our knowledge, these findings provide preliminary clinical observations suggesting that oral SC-BG may enhance human cDC1 activation in vivo and may be relevant to URTI prevention.

SC-BG intake also maintained s-IgA levels in the posterior oropharyngeal wall and nasopharynx and lysozyme levels in the saliva. Secretory IgA (s-IgA) is a major mucosal antibody that prevents pathogens, such as viruses and bacteria, from adhering to mucosal surfaces [42]. Lysozyme, an antimicrobial peptide, degrades peptidoglycan in bacterial cell walls, thereby strengthening innate immunity [43]. Anti-β-glucan antibodies are detected in the sera and saliva of healthy subjects and play a role in defense against fungal infections. SC-BG could stimulate cDC1 in the intestinal mucosa, leading to the maintenance of and increase in IgA production in the nasal cavity and pharynx via common mucosal immune system. The antibody titers are likely to be influenced by seasonal effects. However, the anti-β-glucan antibody titers decreased in the placebo group, contrasting with their maintenance or increase in the SC-BG group, suggesting a beneficial effect of SC-BG intake. A previous clinical study in children with chronic respiratory disease also reported a significant increase in salivary lysozyme levels following SC-BG intake [44]. During winter, when the present study was conducted, mucosal immune defenses are known to decline due to cold and dry environmental conditions [45]. Thus, our findings suggest that SC-BG supports local immune responses in the throat and nasal mucosa, helping maintain barrier function against pathogens when immune defenses are often reduced.

Participants in the SC-BG group also experienced significantly fewer cumulative days with URTI-related symptoms, including local symptoms (runny nose, nasal congestion, sore throat, pain on swallowing, sneezing, cough, and sputum) and systemic symptoms (general fatigue and headache). These results align with those of previous meta-analyses, which demonstrate that yeast β-glucan supplementation reduces both the incidence and severity of URTI symptoms [46]. This improvement may reflect SC-BG-mediated activation of cDC1, which could influence both local mucosal and systemic immune responses.

After oral intake, SC-BG is taken up by gastrointestinal macrophages and transported to the spleen, lymph nodes, and bone marrow [47]. Dendritic cells can receive antigens via M cells or by directly extending dendrites through epithelial layers to capture dietary components and microbes [48,49]. In this study, the activation of circulating cDC1 suggests that SC-BG may have been processed by intestinal macrophages, released as antigenic fragments, and subsequently captured by cDC1. Alternatively, SC-BG may have been directly sampled by mucosal cDC1. Through either mechanism, activated cDC1 can induce both systemic and mucosal immune responses, reducing the occurrence of URTI-related symptoms.

This study has several limitations. First, the sample size was relatively small (*n* = 39), and the study population consisted of healthy adults, which may limit the generalizability of the findings to other populations, including individuals with underlying conditions or different age groups. Second, although multiple mucosal immune markers, such as secretory IgA and salivary lysozyme, were assessed, these biomarkers represent surrogate indicators of mucosal immune status and do not directly reflect resistance to specific infections. In addition, upper respiratory tract symptoms were captured using a daily self-reported diary with a short recall period, which likely helped reduce recall bias. However, because these symptom data were based solely on participant self-report and were not clinically adjudicated or laboratory-confirmed, subjective variability and potential misclassification of cold-like episodes cannot be excluded. Finally, while mechanistic in vitro analyses were performed, the present study was not designed to establish causal pathways linking dietary β-1,3/1,6-glucan intake to clinical outcomes. Larger and longer-term studies are therefore warranted to further clarify the role of dietary polysaccharides in supporting mucosal immune health under diverse real-world conditions. Further research should evaluate whether SC-BG provides protective effects against specific viral infections, such as influenza or COVID-19, and assess its efficacy in populations with underlying conditions.

## 5. Conclusions

This study suggests that dietary β-1,3/1,6-glucan from baker’s yeast may support upper respiratory mucosal immune health in healthy adults. In a randomized, double-blind, placebo-controlled trial, dietary β-1,3/1,6-glucan intake was associated with the maintenance of secretory IgA levels in the posterior oropharyngeal wall and nasopharynx, increased salivary lysozyme concentrations, and a reduced burden of self-perceived upper respiratory tract symptoms. Further studies are warranted to clarify how dietary polysaccharides contribute to the maintenance of mucosal immune health and immune resilience under diverse environmental and lifestyle conditions.

## Figures and Tables

**Table 1:** Nutritional content of test samples (per daily serving).

		Placebo	SC-BG
		Capsules	Capsules
Energy	kcal	2.6	2.7
Protein	g	<0.1	<0.1
Fat	g	<0.1	<0.1
Sugar	g	0.6	0.6
Sodium	mg	<0.1	<0.1

**Table 2:** Summaries of MFI values for insoluble binding and laminarin-equivalent values for soluble fractions.

Saccharides	Supplier	Insoluble (MFI)	Soluble (µg Laminarin Equivalent/100 µg)
SC-BG	Kerry	18,084 ± 8078 ***	9.78 ± 1.97 ^†††^
Glucan from baker’s yeast	Sigma-Aldrich	19,462 ± 2497 ***	7.43 ± 4.36 ^†††^
Zymosan	Invivogen	4188 ± 1252	7.58 ± 1.32 ^†††^
Zymosan A from *Saccharomyces cerevisiae*	Sigma-Aldrich	5124 ± 810	7.96 ± 0.99 ^†††^
Zymosan A	Fujifilm Wako	30,070 ± 6852 ***	2.76 ± 1.57 ^†††^
depleted zymosan	Invivogen	2510 ± 893	0.00 ± 0.00 ^†††^
Curdlan	Sigma-Aldrich	816 ± 887	0.00 ± 0.00 ^†††^
β-1,3-Glucan from *Euglena gracilis*	Sigma-Aldrich	1773 ± 1097	0.23 ± 0.20 ^†††^
Laminarin from *Laminaria digitata*	Sigma-Aldrich	161± 244	235.76 ± 10.75
β-1,3, 1,4-Glucan from barley	Sigma-Aldrich	236 ± 451	0.09 ± 0.01 ^†††^
Fucoidan	Fujifilm Wako	125 ± 73	0.00 ± 0.00 ^†††^
Cellulose	Sigma-Aldrich	153 ± 170	0.00 ± 0.00 ^†††^

Values are expressed as mean ± standard deviation for quantitative data. In insoluble parts, *n* = 5 from 3 independent experiments. All samples were compared by Dunnet’ test vs. Cellulose. ***, *p* < 0.001. In soluble parts, *n* = 4 from 2 independent experiments. All samples were compared by Dunnet’ test vs. Laminarin. ^†††^, *p* < 0.001.

**Table 3:** Baseline characteristics of participants (FAS).

		Placebo Group	SC-BG Group
Variable		(*n* = 20)	(*n* = 19)
Sex	female	13 (65.0%)	11 (57.9%)
	male	7 (35.0%)	8 (42.1%)
Age	years	44.5 ± 10.7	43.8 ± 10.1
Height	cm	161.7 ± 9.0	165.0 ± 8.5
Weight	kg	61.4 ± 13.5	61.8 ± 10.1
Body mass index	kg/m^2^	23.3 ± 4.0	22.7 ± 3.6

Values are expressed as mean ± standard deviation for quantitative data and *n* (%) for categorical data (FAS).

**Table 4:** Mean Fluorescence Intensity (MFI) of CD40, CD80 and CD86.

			Base Line		Week 8	
				*p*-Value		*p*-Value
cDC1	CD40	Placebo	3970 ± 1786	0.277	3148 ± 1746 *	0.447
		SC-BG	3318 ± 1905		3533 ± 1338	
	CD80	Placebo	−925 ± 1200	0.933	−1200 ± 1748	0.005
		SC-BG	−860 ± 2433		−422 ± 791	
	CD86	Placebo	740 ± 220	0.756	696 ± 197	0.573
		SC-BG	765 ± 268		655 ± 243	
cDC2	CD40	Placebo	1126 ± 1042	0.693	1010 ± 962	0.531
		SC-BG	1068 ± 991		924 ± 870	
	CD80	Placebo	61 ± 50	0.241	83 ± 77	0.878
		SC-BG	114 ± 88		89 ± 65	
	CD86	Placebo	1293 ± 1284	0.685	1397 ± 1390 *	0.214
		SC-BG	1336 ± 1429		1285 ± 1258	

Statistical significance for the comparison between the placebo and SC-BG groups is presented numerically in the “*p*-value” column. Intra-group significance (comparison of weeks 8 versus baseline within the same group) is denoted by asterisks: * p < 0.05.

**Table 5:** Changes in s-IgA Levels in Saliva, Oropharyngeal Swabs, and Nasopharyngeal Swabs and Lysozyme Levels in Saliva from Baseline.

			Base Line (Mean ± SD)		Week 8 (Mean ± SD)		Week 12 (Mean ± SD)		Week 8 (Δ Mean ± SD)		Week 12 (Δ Mean ± SD)	
				*p*-Value		*p*-Value		*p*-Value		*p*-Value		*p*-Value
s-IgA	Saliva	Placebo	213 ± 104	0.718	224 ± 101	0.835	246.5 ± 114.0	0.264	11.0 ± 77.6	0.445	22.6 ± 62.7	0.218
		SC-BG	227 ± 140		217 ± 104		308.5 ± 209.8 *		−10.1 ± 93.1		55.9 ± 100	
	Nasal	Placebo	0.60 ± 0.23	0.079	0.55 ± 0.27	0.180	0.44 ± 0.22 **	0.243	−0.04 ± 0.26	0.531	−0.16 ± 0.24	0.015
		SC-BG	0.54 ± 0.21		0.45 ± 0.22		0.53 ± 0.23		−0.09 ± 0.20		−0.01 ± 0.21	
	Throat	Placebo	1.82 ± 1.35	0.108	0.88 ± 0.85 **	0.556	0.83 ± 0.70 **	0.175	−0.93 ± 1.08	0.044	−0.99 ± 1.23	0.461
		SC-BG	1.26 ± 0.63		1.05 ± 0.92		0.53 ± 0.63 **		−0.21 ± 1.09		−0.73 ± 0.90	
Lysozyme	Saliva	Placebo	0.91 ± 0.99	0.657	0.58 ± 0.56 *	0.159	0.27 ± 0.33 **	0.001	−0.33 ± 0.62	0.560	−0.64 ± 0.92	0.452
		SC-BG	1.04 ± 0.74		0.84 ± 0.56		0.61 ± 0.37 *		−0.20 ± 0.77		−0.43 ± 0.83	

Statistical significance for the comparison between the placebo and SC-BG groups is presented numerically in the “*p*-value” column. Intra-group significance (comparison of weeks 8 and 12 versus baseline within the same group) is denoted by asterisks: * *p* < 0.05 and ** *p* < 0.01.

**Table 6:** Changes in IgA, NK cell activity, neutrophil phagocytosis, and CRP levels in blood from baseline.

		Base Line (Mean ± SD)		Week 12 (Mean ± SD)		Week 12 (Δ Mean ± SD)	
			*p*-Value		*p*-Value		*p*-Value
IgA	Placebo	238 ± 89	0.950	250 ± 95 **	0.880	11.9 ± 14.7	0.620
	SC-BG	239 ± 86		254 ± 89 **		14.6 ± 19.2	
NK cell activity	Placebo	38.3 ± 12.3	0.931	35.6 ± 15.6	0.796	−2.65 ± 12.0	0.817
	SC-BG	37.9 ± 13.2		34.3 ± 15.1		−3.58 ± 12.8	
Neutrophil phagocytosis	Placebo	92.5 ± 6.6	0.648	96.2 ± 1.8 *	0.305	3.69 ± 7.22	0.941
	SC-BG	93.3 ± 4.8		96.9 ± 2.3 *		3.53 ± 5.91	
CRP	Placebo	0.09 ± 0.14	0.992	0.11 ± 0.14	0.848	0.02 ± 0.12	0.681
	SC-BG	0.09 ± 0.23		0.09 ± 0.24		0.01 ± 0.04	

Statistical significance for the comparison between the placebo and SC-BG groups is presented numerically in the “*p*-value” column. Intra-group significance (comparison of weeks 12 versus baseline within the same group) is denoted by asterisks: * *p* < 0.05 and ** *p* < 0.01.

**Table 7:** Comparison of Cumulative Days of Each Symptom.

		Cumulative Number of Days from Weeks 0 to 8			Cumulative Number of Days from Weeks 0 to 12		
		Without Symptom	With Symptom	*p*-Value	Without Symptom	With Symptom	*p*-Value
Runny nose	Placebo	233	211	0.001	1374	286	<0.001
	SC-BG	919	145		1376	201	
Nasal congestion	Placebo	983	137	<0.001	1462	198	<0.001
	SC-BG	983	81		1470	107	
Sore throat	Placebo	1057	63	<0.001	1587	73	<0.001
	SC-BG	1045	18		1552	24	
Pain on swallowing	Placebo	1080	40	<0.001	1618	42	<0.001
	SC-BG	1055	8		1568	8	
Sneezing	Placebo	982	138	<0.001	1451	209	<0.001
	SC-BG	1019	45		1519	58	
Cough	Placebo	1061	59	<0.001	1572	88	<0.001
	SC-BG	1053	11		1554	23	
Phlegm (sputum)	Placebo	1038	82	<0.001	1542	118	<0.001
	SC-BG	1057	7		1563	14	
General fatigue (malaise)	Placebo	1017	103	<0.001	1532	128	<0.001
	SC-BG	1018	46		1518	59	
Muscle pain	Placebo	1085	35	0.826	1606	54	0.892
	SC-BG	1028	36		1528	49	
Joint pain	Placebo	1105	15	0.031	1644	16	<0.001
	SC-BG	1035	29		1535	42	
Headache	Placebo	1032	88	0.108	1532	128	0.005
	SC-BG	1000	64		1495	82	
Loss of appetite	Placebo	1094	26	0.005	1624	36	0.217
	SC-BG	1056	8		1553	24	
Chills	Placebo	1106	14	0.475	1636	24	0.036
	SC-BG	1055	9		1567	10	

## Data Availability

Data generated or analyzed during the study are available from the corresponding author upon reasonable request.

## Supplementary Materials

The following supporting information can be downloaded at: https://www.mdpi.com/article/10.3390/nu18060961/s1. These include: Table S1: Details of Antibodies Used in Flow Cytometry. Table S2: Changes in β-Glucan Antibody Titers in Blood, Saliva, Nasopharyngeal Swabs, and Oropharyngeal Swabs. Table S3: Comparison of Cumulative Symptom Days (No Symptoms vs. Moderate to Severe). Table S4: Comparison of Cumulative Symptom Days (No Symptoms vs. Severe). Table S5: Summary of SF-8 QOL Scores. Figure S1: Gating strategy for the identification and activation analysis of cDCs. Figure S2: Gating strategy for the identification of insoluble particles in polysaccharide reagents. Figure S3: Gating strategy for the identification of cDC1 and cDC2.

## Abbreviations

The following abbreviations are used in this manuscript:

SC-BG	β-1,3/1,6-glucan derived from *Saccharomyces cerevisiae*
BG	β-1,3-D-glucan
cDC	Conventional dendritic cell
cDC1	Conventional dendritic cell subtype 1
cDC2	Conventional dendritic cell subtype 2
pDC	Plasmacytoid dendritic cell
hPBMC	Human peripheral blood mononuclear cell
MFI	Mean fluorescence intensity
s-IgA	Secretory immunoglobulin A
NK	Natural killer (cell)
CRP	C-reactive protein
URTI	Upper respiratory tract infection
TNF-α	Tumor necrosis factor alpha
FAS	Full Analysis Set
CONSORT	Consolidated Standards of Reporting Trials
PF	Physical Functioning
MCS	Mental Component Summary
RE	Role Functioning
MH	Mental Health
