# Supplementary material for: Dietary β-1,3/1,6-Glucan from Baker’s Yeast Supports Upper Respiratory Mucosal Immune Health in Healthy Adults: Evidence from a Randomized, Double-Blind, Placebo-Controlled Trial
Source: Nutrients. 2026 Mar 18;18(6):961. doi: 10.3390/nu18060961 (PMC13028741; doi:10.3390/nu18060961)
Supplement: Supplementary file 1 [file nutrients-18-00961-s001.zip › nutrients-4149929-supplementary_figures.pdf]

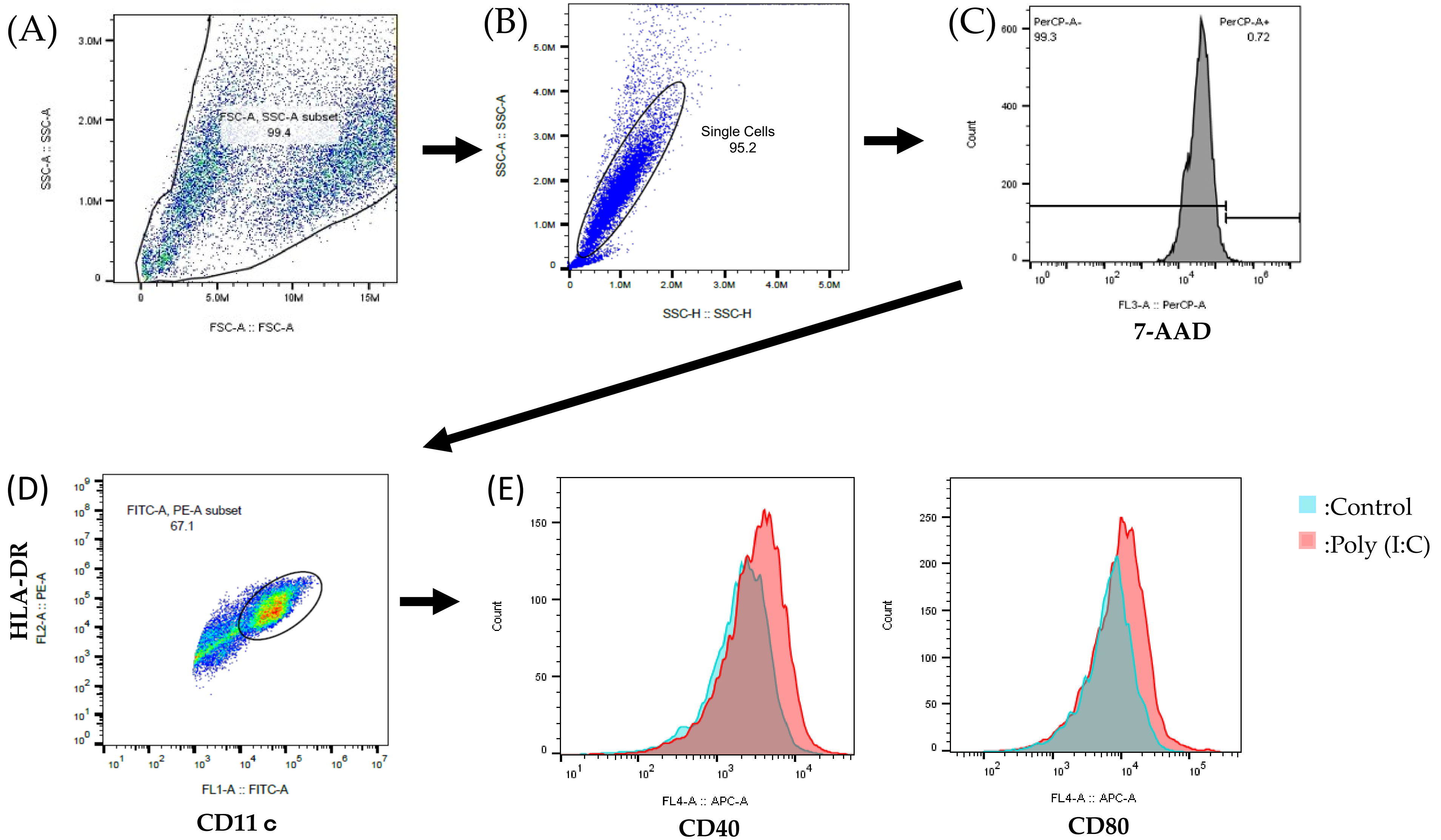

Supplementary Figure 1. Gating strategy for the identification and activation analysis of cDCs.
Representative flow cytometry plots illustrate the gating hierarchy of conventional dendritic cells (cDCs). (A) Initial gating was performed using forward scatter area (FSC-A) and side scatter area (SSC-A) to exclude debris. (B) Single cells were identified by employing side scatter height (SSC-H) versus SSC-A to eliminate doublets. Dead cells were excluded based on 7-AAD staining, with 7-AAD-negative cells defined as viable. (D) cDCs were characterized as the CD11c+ HLA-DR+ population. (E) Within the cDC gate, the expression levels of activation markers CD40 and CD80 were assessed. The plots display the percentage of cells in each gated population for both the control and poly (I:C) groups.

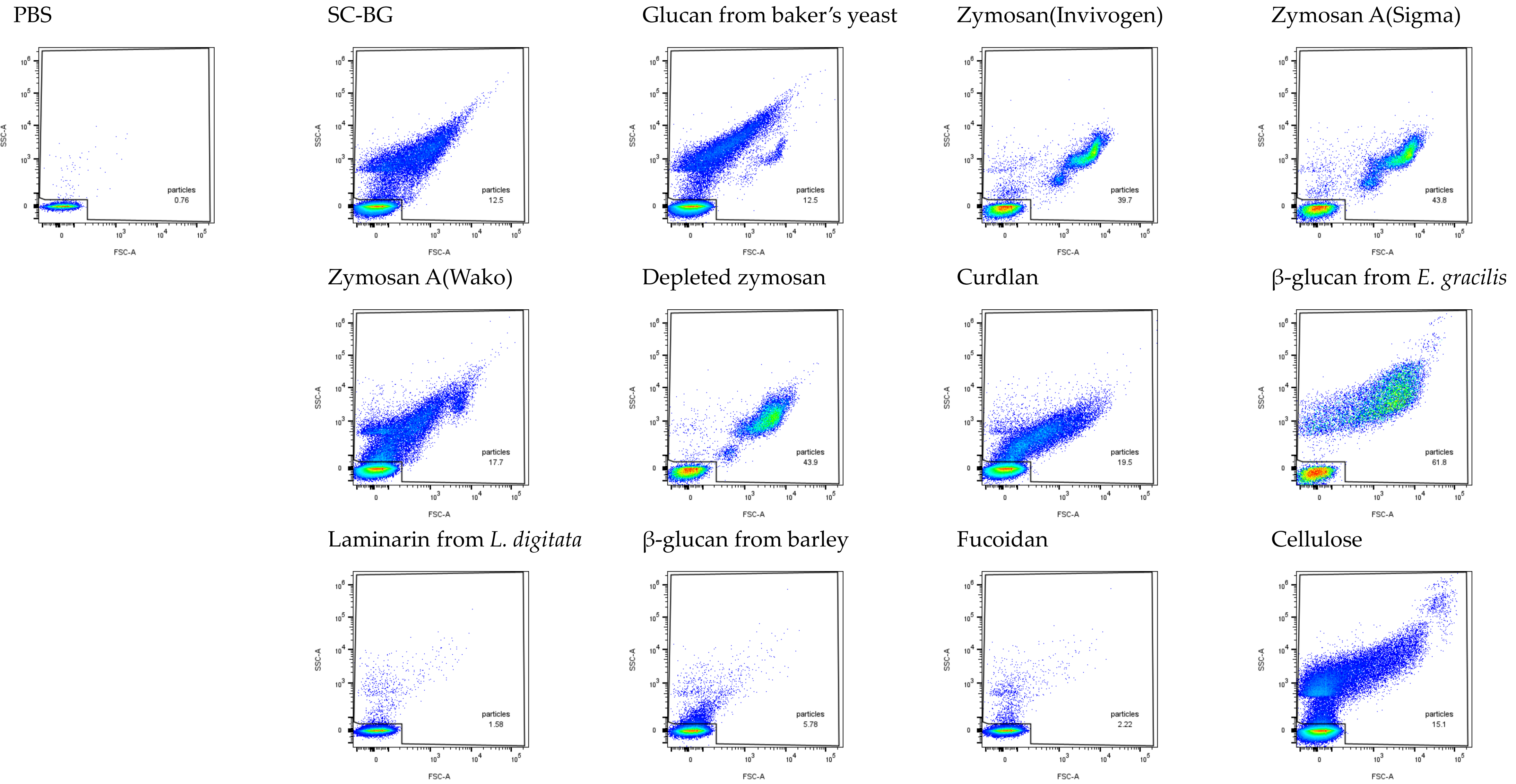

Supplementary Figure 2. Gating strategy for the identification of insoluble particles in polysaccharide reagents.
Representative flow cytometry plots illustrate the gating of particles within each polysaccharide reagent. The FSC-SSC gates were established using the plot of PBS to exclude debris.

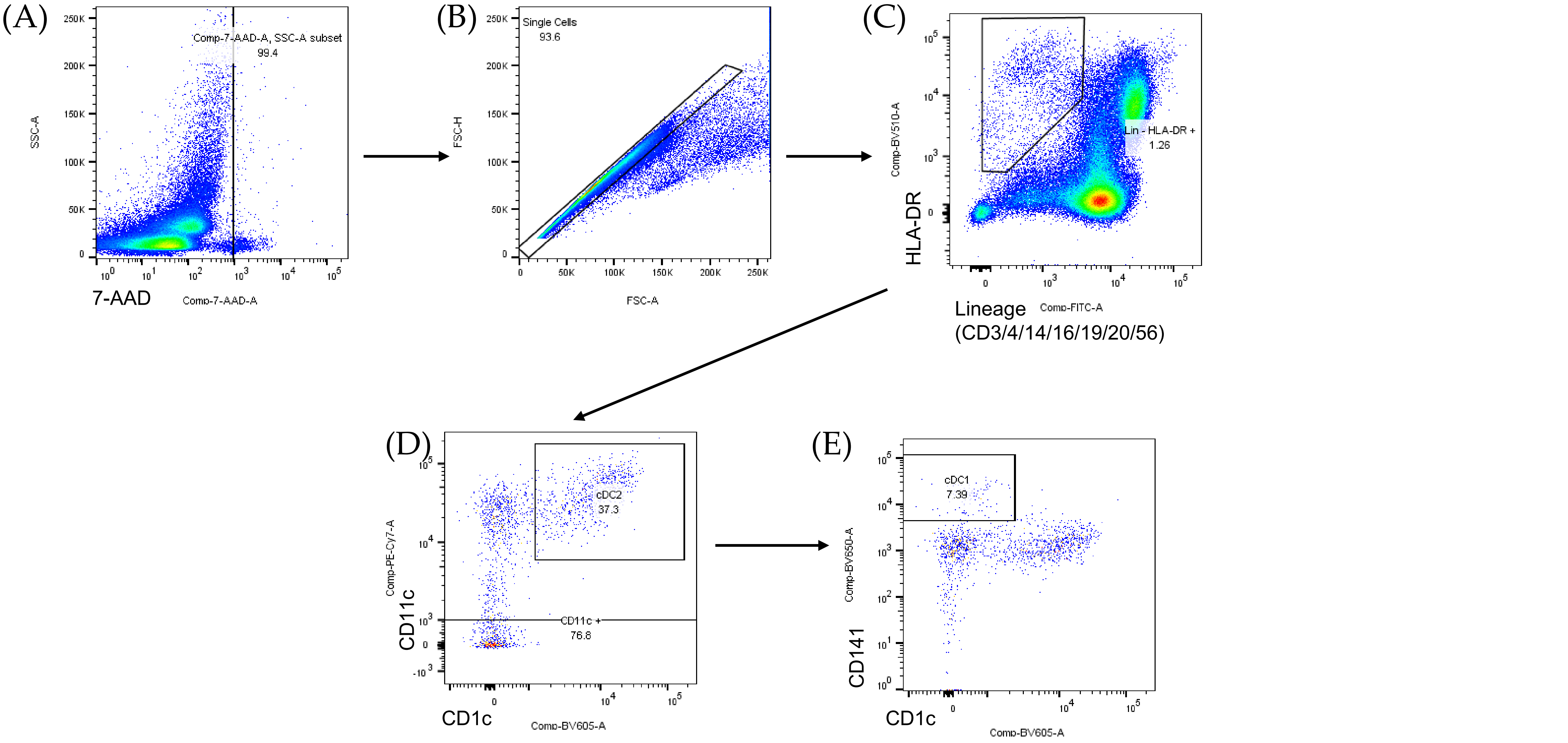

Supplementary Figure 3. Gating strategy for the identification of cDC1 and cDC2.
Representative flow cytometry plots illustrate the gating hierarchy for cDC1 and cDC2 cells. (A) Dead cells were excluded based on 7-AAD staining, with 7-AAD-negative cells defined as live cells. (B) Single cells were identified using FSC-H and FSC-A to exclude doublets. (C) Non-dendritic cells (DCs) were excluded by Lineage, where Lineage-positive cells included T cells, B cells, NK cells, monocytes, macrophages, and neutrophils, and HLA-DR. (D) cDC2 cells were characterized by the co-expression of CD11c and CD1c markers. (E) cDC1 cells were identified as CD11c-gated, CD141-positive cells.
