# Supplementary material for: Dietary β-1,3/1,6-Glucan from Baker’s Yeast Supports Upper Respiratory Mucosal Immune Health in Healthy Adults: Evidence from a Randomized, Double-Blind, Placebo-Controlled Trial
Source: Nutrients. 2026 Mar 18;18(6):961. doi: 10.3390/nu18060961 (PMC13028741; doi:10.3390/nu18060961)
Supplement: Supplementary file 1 [file nutrients-18-00961-s001.zip › nutrients-4149929-supplementary_tables.pdf]

Supplementary Table 1 Details of Antibodies Utilized in Flow Cytometry

Fluorescence	Antigen	Clone	Isotype Ctrl	Clone	Supplier
FITC	Lineage (CD3, CD14, CD16, CD19, CD20, CD56)	UCHT1, HCD14, 3G8, HIB19; 2H7, HCD56	Mouse IgG1<br>Mouse IgG2b	MOPC-21<br>MPC-11	Biolegend
PE	Dectin-1	REA515	Human IgG1	REA293	Miltenyi
PE/Cy7	CD11c	Bu15	Mouse IgG1	MOPC-21	Biolgend
APC	CD40	5C3	Mouse IgG1	MOPC-21	Biolgend
BV421	CD80	2D10	Mouse IgG2a	MOPC-173	Biolgend
BV510	HLA-DR	L243	Mouse IgG2b	MPC-11	Biolgend
BV605	CD1c	L161	Mouse IgG1	MOPC-21	Biolgend
BV650	CD141	1A4	Mouse IgG1	X40	BD
BV785	CD86	IT2.2	Mouse IgG1	MOPC-21	Biolgend

Supplementary Table 2 Changes in β Glucan Antibody Titers in Blood, Saliva, Nasopharyngeal Swabs, and Oropharyngeal Swabs from Baseline

			Base line (Mean ± SD)	*P*-value	Week 8 (Mean ± SD)	*P*-value	Week 12 (Mean ± SD)	*P*-value	Week 8 (Δ Mean ± SD)	*P*-value	Week 12 (Δ Mean ± SD)	*P*-value
anti-BG IgA	Serum	Placebo	0.41 ± 0.25		0.39 ± 0.25		-		-0.02 ± 0.09		-	
				0.142		0.246		-		0.365		-
		SC-BG	0.54 ± 0.27		0.49 ± 0.27*		-		-0.05 ± 0.10		-	
	Saliva	Placebo	0.54 ± 0.32		0.69 ± 0.38**		-		0.15 ± 0.23		-	
				0.549		0.698		-		0.958		-
		SC-BG	0.60 ± 0.34		0.73 ± 0.37**		-		0.15 ± 0.19		-	
	Nasal	Placebo	0.44 ± 0.31		0.51 ± 0.25		0.51 ± 0.30		0.07 ± 0.24		0.06 ± 0.25	
				0.558		0.769		0.961		0.290		0.496
		SC-BG	0.39 ± 0.21		0.54 ± 0.33**		0.50 ± 0.23**		0.15 ± 0.21		0.11 ± 0.16	
	Throat	Placebo	0.49 ± 0.45		0.17 ± 0.23**		0.15 ± 0.23**		-0.32 ± 0.40		-0.34 ± 0.38	
				0.525		0.058		0.747		0.029		0.419
		SC-BG	0.41 ± 0.33		0.47 ± 0.60		0.19 ± 0.45		0.06 ± 0.62[†]		-0.22 ± 0.52	
anti-BG IgG	Serum	Placebo	0.77 ± 0.32		0.69 ± 0.24*		-		-0.08 ± 0.16		-	
				0.217		0.410		-		0.207		-
		SC-BG	0.66 ± 0.24		0.63 ± 0.19		-		-0.02 ± 0.10		-	

Statistical significance for the comparison between the placebo and SC-BG groups is presented numerically in the "*P*-value" column. Intra-group significance (comparison of Weeks 8 and 12 versus Baseline within the same group) is denoted by asterisks: *$P < 0.05$ and **$P < 0.01$.

Supplementary Table 3 Comparison of the Cumulative Days of Each Symptom (No symptoms vs. Moderate to Severe symptoms))

		Cumulative number of days from Weeks 0 to 8			Cumulative number of days from Weeks 0 to 12		
		Without Symptom	With Symptom	*P*-value	Without Symptom	With Symptom	*P*-value
Runny nose	Placebo	909	22	0.975	1374	30	0.717
	SC-BG	919	21		1376	34	
Nasal congestion	Placebo	983	28	0.006	1462	36	0.002
	SC-BG	983	10		1470	13	
Sore throat	Placebo	1057	27	< 0.001	1587	27	< 0.001
	SC-BG	1045	2		1552	4	
Pain on swallowing	Placebo	1080	17	< 0.001	1618	17	< 0.001
	SC-BG	1055	1		1568	1	
Sneezing	Placebo	982	4	1.000	1451	8	0.726
	SC-BG	1019	5		1519	6	
Cough	Placebo	1061	7	0.785	1572	7	0.789
	SC-BG	1053	5		1554	5	
Phlegm (sputum)	Placebo	1038	11	0.009	1542	11	0.009
	SC-BG	1057	1		1563	1	
General fatigue (malaise)	Placebo	1017	2	0.115	1532	3	0.038
	SC-BG	1018	8		1518	12	
Muscle pain	Placebo	1085	4	1.000	1606	5	0.260
	SC-BG	1028	4		1528	10	
Joint pain	Placebo	1105	0	0.974	1644	0	0.117
	SC-BG	1035	1		1535	4	
Headache	Placebo	1032	9	0.008	1532	14	0.053
	SC-BG	1000	25		1495	27	
Loss of appetite	Placebo	1094	0	-	1624	0	-
	SC-BG	1056	0		1553	0	
Chills	Placebo	1106	1	0.201	1636	1	-
	SC-BG	1055	5		1567	5	

Statistical significance for the comparison between the placebo and SC-BG groups is presented numerically in the "*P*-value" column.

Supplementary Table 4 Comparison of the Cumulative Days of Each Symptom (No symptoms vs. Severe symptoms))

		Cumulative number of days from Weeks 0 to 8			Cumulative number of days from Weeks 0 to 12		
		Without Symptom	With Symptom	*P*-value	Without Symptom	With Symptom	*P*-value
Runny nose	Placebo	909	0	0.137	1374	1	0.373
	SC-BG	919	4		1376	4	
Nasal congestion	Placebo	983	0	0.134	1462	0	0.135
	SC-BG	983	4		1470	4	
Sore throat	Placebo	1057	7	0.025	1587	7	0.025
	SC-BG	1045	0		1552	0	
Pain on swallowing	Placebo	1080	0	-	1618	0	-
	SC-BG	1055	0		1568	0	
Sneezing	Placebo	1080	0	-	1618	0	0.982
	SC-BG	1055	0		1568	0	
Cough	Placebo	1061	2	0.483	1572	2	0.485
	SC-BG	1053	0		1554	0	
Phlegm (sputum)	Placebo	1038	1	0.993	1542	1	0.995
	SC-BG	1057	0		1563	0	
General fatigue (malaise)	Placebo	1017	0	1.000	1532	0	0.997
	SC-BG	1018	1		1518	1	
Muscle pain	Placebo	1085	2	0.504	1606	2	1.000
	SC-BG	1028	0		1528	2	
Joint pain	Placebo	1105	0	-	1644	0	-
	SC-BG	1035	0		1535	0	
Headache	Placebo	1032	4	0.964	1532	7	0.807
	SC-BG	1000	5		1495	5	
Loss of appetite	Placebo	1094	0	-	1624	0	-
	SC-BG	1056	0		1553	0	
Chills	Placebo	1106	0	-	1636	0	-
	SC-BG	1055	0		1567	0	

Statistical significance for the comparison between the placebo and SC-BG groups is presented numerically in the "*P*-value" column.

Supplementary Table 5 Summary of SF-8

		Base line (Mean ± SD)	*P*-value	Week 8 (Mean ± SD)	*P*-value	Week 12 (Mean ± SD)	*P*-value
PCS	Placebo	51.3 ± 3.5	0.684	50.6 ± 6.4	0.888	50.2 ± 5.4	0.440
	SC-BG	51.8 ± 3.8		51.7 ± 3.4		51.7 ± 4.1	
MCS	Placebo	48.7 ± 5.3	0.509	50.1 ± 4.6	0.168	50.0 ± 6.1	0.292
	SC-BG	49.6 ± 5.3		52.3 ± 3.6 **		51.9 ± 5.6	
PF	Placebo	52.1 ± 3.1	0.677	50.8 ± 4.5 *	0.128	51.7 ± 3.5	0.872
	SC-BG	52.4 ± 3.4		52.9 ± 2.9		51.6 ± 2.8	
RP	Placebo	51.1 ± 4.5	0.690	50.9 ± 4.8	0.222	50.1 ± 7.3	0.581
	SC-BG	51.6 ± 4.6		52.6 ± 3.4		52.0 ± 3.9	
BP	Placebo	50.3 ± 7.2	0.321	50.6 ± 9.2	0.343	49.9 ± 8.4	0.080
	SC-BG	52.3 ± 5.4		53.6 ± 7.2		54.0 ± 8.4	
GH	Placebo	52.8 ± 4.3	0.311	53.1 ± 5.4	0.749	53.3 ± 3.6	0.269
	SC-BG	54.3 ± 4.7		54.1 ± 3.4		54.7 ± 4.8	
VT	Placebo	52.0 ± 4.3	0.975	53.1 ± 4.4	0.599	51.6 ± 4.4	0.230
	SC-BG	51.9 ± 4.9		52.4 ± 4.5		53.1 ± 4.6	
SF	Placebo	51.1 ± 5.4	0.974	52.1 ± 4.5	0.507	51.9 ± 5.4	0.532
	SC-BG	50.6 ± 6.9		53.1 ± 3.6		52.9 ± 4.8	
RE	Placebo	49.9 ± 5.9	0.892	50.5 ± 5.5	0.132	50.3 ± 6.0	0.117
	SC-BG	50.5 ± 4.9		53.2 ± 2.9 *		53.0 ± 4.0 *	
MH	Placebo	49.2 ± 5.9	0.268	49.9 ± 4.5	0.009	50.3 ± 6.2	0.257
	SC-BG	51.3 ± 4.9		53.5 ± 2.8 *		52.5 ± 5.0	

Statistical significance for the comparison between the placebo and SC-BG groups is presented numerically in the "p-value" column. Intra-group significance (comparison of Weeks 8 and 12 versus Baseline within the same group) is denoted by asterisks: * $P < 0.05$ and ** $P < 0.01$
